# Pesticide metabolite and oxidative stress in male farmers exposed to pesticide

**DOI:** 10.1186/s40557-017-0162-3

**Published:** 2017-02-28

**Authors:** Kang Myoung Lee, Sang-Yoo Park, Kyungsuk Lee, Sung-Soo Oh, Sang Baek Ko

**Affiliations:** 1 Department of Medicine, The Graduate School, Yonsei University, Seoul, South Korea; 2 Department of Otolaryngology Head and Neck Surgery, Wonju College of Medicine, Yonsei Unversity, Seoul, South Korea; 3 National Academy of Agricultural Science, Rural Development Administration, Jeonju, South Korea; 4 Department of Preventive Medicine and Institute of Occupational and Environmental Medicine, Wonju College of Medicine, Yonsei Unversity, Seoul, South Korea

**Keywords:** Oxidative stress, Pesticide metabolite, Pesticide exposure

## Abstract

**Background:**

The objective of this study was to measure malondialdehyde (MDA) and isoprostane which has been used as an index of lipid injury, 8-hydroxy-2′-deoxyguanosine (8-OHdG), which has been used as an index of DNA damage, and dialkyl-phosphate (DAP), which has been used to quantify pesticide exposure, and to investigate the relationship between pesticide exposure and oxidative stress.

**Methods:**

This study was a cross-sectional study that evaluated 84 male farmers exposure to pesticide. In this study, 8-OHdG, isoprostane, and MDA were measured as oxidative stress indices, and dialkyl-phosphate (dimethylphosphate(DMP), diethylphosphate(DEP), dimethylthiophosphate(DMTP), and diethylthiophosphate (DETP)) excreted in the urine was also measured to evaluate pesticide exposure. A linear regression analysis was performed to investigate the relationship between pesticide metabolites, and oxidative stress biomarkers.

**Results:**

A Correlation analysis was performed for pesticide exposure month (PEI), cumulative exposure index (CEI), and DAP as well as the concentration of the oxidative stress biomarkers. The PEM significantly and positively correlated to the levels of 8-OHdG, isoprostane, CEI, and DMP. CEI showed a correlation to 8-OHdG and PEM. DMP, DEP, and DETP showed a positive correlation to 8-OHdG, isoprostane, and MDA. A correlation analysis was adjusted some demographic characteristics, such as age, smoking, drinking, and exercise to determine the relationship between pesticide exposure and oxidative stress. The 8-OHdG, isoprostane, and MDA levels were significantly related to the DMP (ß = 0.320), DEP (ß = 0.390), and DETP (ß = 0.082); DMP (ß = 0.396), DEP (ß = 0.508), and DETP (ß = 0.504); and DMP (ß = 0.432), DEP (ß = 0.508), and DETP (ß = 0.329) levels, respectively.

**Conclusions:**

The concentration between oxidative stress biomarkers and the pesticide metabolite were a positive correlation. Indicators of oxidative stress was associated with a pesticide metabolite DMP, DEP, and DETP. Therefore, Pesticide exposure and oxidative stress were relevant.

## Background

Pesticides are agricultural chemicals that protect crops and stored products, partially by exterminating harmful insects. Pesticides can be classified into several different types according to their purpose, such as insecticides, fungicides, herbicides, acaricides, rodenticides, nematicides, and plant growth regulators. Pesticides can also be classified according to chemical composition (organophosphates, organochlorines, carbamates, pyrethroids, sulfur, and urea) or powder type (emulsifier, wettable powder, soluble powder, powder, particle powder, and smoke powder).

Pesticides are extensively used throughout the world and influence the human body through long-term exposure [1, 2]. A previous study on the chronic toxicity of pesticides showed that the cognitive ability and exercise performance of children in areas sprayed with pesticides were significantly decreased compared with those of children from non-sprayed areas [3]. Pesticide exposure was also found to be significantly related to lung cancer, pancreatic cancer, colon cancer, rectal cancer, leukemia, hematologic malignancy, lymphoma, multiple myeloma, bladder cancer, prostate cancer, brain tumor, and skin cancer [4]. In particular, it has been reported that organophosphorus pesticides such as crotoxyphos, dichlorvos, famphur, diazinon, fonofos, malathion, and phorate, increased the risk of leukemia, non-Hodgkin’s lymphoma, and prostate cancer. In addition, chlorpyrifos resulted in increased lung cancer rates upon direct exposure [5].

Previous studies performed in humans and animals indicated that organophosphorus pesticides particularly caused such oxidative stress [6–9]. Organochlorine, carbamate, and pyrethroid pesticides have also show toxicity [10, 11] and oxidative stress may cause acute and chronic inflammatory diseases [12].

Malondialdehyde(MDA) and 8-iso-prostaglandin (isoprostane) are used to evaluate lipid injury, 8-hydroxy-2′-deoxyguanosine (8-OHdG) is used to evaluate protein and lipid injury. Pesticide exposure can thus be usually measured by evaluating the cholinesterase activity in the blood by sampling the blood before and after such exposure. However, this method is qualitative and not quantitative. A different method for evaluating pesticide exposure is to quantify the levels of urinary dialkyl-phosphate (DAP), which is an organophosphorus metabolite. This method shows excellent sensitivity for biological monitoring and can be used to quantitatively evaluate the pesticide exposure to the human body. The DAP detected in urine is usually metabolized as dimethylphosphate (DMP), diethylphosphate (DEP), dimethylthiophosphate (DMTP), and diethylthiophosphate (DETP). The amount of pesticide exposure can thus be quantitatively evaluated by analyzing the amount of these metabolites.

The objective of this study was to measure malondialdehyde (MDA) and isoprostane which has been used as an index of lipid injury, 8-hydroxy-2′-deoxyguanosine (8-OHdG), which has been used as an index of DNA damage, and dialkyl-phosphate (DAP), which has been used to quantify pesticide exposure, and to investigate the relationship between pesticide exposure and oxidative stress.

## Methods

### Selection of Subjects

This study was a cross-sectional study of 104 male farmers exposure to pesticide who were selected from 126 male farmers among 290 people living in G Li of Wonju city, South Korea during the period May to August 2011. In total, 11 subjects who had stomach cancer, liver cancer, cancer of the large intestine, lung cancer, bladder cancer, type B and C hepatitis, angina pectoris, stroke, and myocardial infarction were excluded. 9 subjects who did not answer the questionnaire were also excluded. Thus, 84 subjects were selected for the urine test to oxidative stress biomarker and pesticide metabolite. The survey focused on general items (e.g., age, smoking, and drinking), pesticide-related items (e.g., spraying of pesticides, duration of spraying pesticides, days of spraying, types of pesticides, amount of pesticides, and method for spraying pesticides), items for observing compliance with regulations related to the spraying of pesticides.

### Oxidative stress biomarker assays

8-OHdG is a promutagenic lesion in DNA that is generated in response to a number of chemicals that induce oxidative stress [13], and it is used as a biomarker of oxidative stress in DNA [14]. The measurement tool was a high-performance liquid chromatography-triple tandem mass detector (HPLC-MS/MS; Agilent 6410, Agilent). 8-OHdG was purchased from Calbiochem (CA, USA), and 2′-deoxyguanosine was purchased from Sigma (St Louis, MO, USA). 15 N5-2′-deoxyguanosine and 5′-triphosphate (15 N5-dGTP) were purchased from Martek (Columbia, MD, USA), and alkaline phosphate was purchased from Roche (Mannheim, Germany). Ammonium acetate and methyl alcohol (MeOH) were purchased from Sigma. Pre-treated urine samples were subjected to solid-phase extraction. The HPLC-MS/MS mass spectrometer was equipped with an Agilent (2.1 × 100 mm × 3.5 μm) column. Ammonium acetate and methyl alcohol were used as the mobile phase with a column flow of 0.16 mL/min. The applied injection volume was 6 μL for the quantification mode (MS SIM mode + MRM mode).

Isoprostane was used to detect lipid peroxidation in urine. The measurement tool was a high-performance liquid chromatography-triple tandem mass detector (HPLC-MS/MS; Agilent 6410, Agilent). The HPLC-MS/MS mass spectrometer was equipped with a PGC (Hypercarb, 5 μm × 150 mm × 1.0) column. Water and acetonitrile with MeOH were used as the mobile phase with a column flow of 60 mL/min. The applied injection volume was 10 μL for the quantification mode (MS SIM mode + MRM mode).

Hydrolyzing 1,1,3,3-tetramethoxypropane (TMP. 99%, Sigma) and 1.0 N HCl generated a MDA standard. Analysis was conducted with HPLC after derivatization with 2,4-DNPH. TMP (165 μl, 1 mmol) and 835 μl 1.0 N HCl were mixed and were diluted ten-fold with 1.0 N HCl. Samples were incubated at 40 °C for 30 min and 100 mM TMP hydrolysate (malondialdehyde, MDA) was added. A final level of MDA produced through hydrolysis was calculated by measuring the optical density at 245 nm and using maximum molar extinction coefficient (ℇ = 13,700). After centrifuging the urine samples at 4000 rpm for 10 min, the supernatant was isolated for analysis. Urine samples of workers were collected in polyethylene bottles from the first urination in the morning. They were kept at -85 °C until further analysis. The urinary MDA levels of each individual will be corrected according to urine creatinine values, which were measured using an automated method based on the Jaffe reaction. Analysis method was used HPLC equipment. Analysis was performed on a HPLC, Waters separation Module Alliance 2695 and Water 2487 Dual ƛ Absorbance detector equipped with a Higgins (4.6 × 250 mm × 5 μm) column. Mobile phase with a column flow of 1 ml/min was used as a acetonitrile (40%) and 20 mM K_2_PO_4_ (60%) in phosphate acid 100 μl. Injection volume was 20 μl of solution into the system and analysed under the following conditions.

### Pesticide exposure index

Exposure to pesticides may occur while transporting, mixing, applying chemicals, through cleaning or repairing equipment. Factors affecting the level of exposure include type of activity (e.g. application, mixing), method of application (e.g. backpack, hand spray, speed sprayer), use of personal protective equipment (PPE) (e.g. gloves, respirators, face-shields, boots or overalls), and personal work habits and hygiene (e.g. changing into clean clothes or taking bath after the use of pesticide). The challenge was to incorporate these exposure modifiers into an estimation of intensity of pesticide exposure. Pesticide exposure index were used to estimate the intensity of exposure to individual pesticides using the intensity level. In the literature Dosemeci [15], there was applied to modify the intensity levels.

$$ *\ \mathrm{Intensity}\ \mathrm{level} = \left(\mathrm{mixing}\ \mathrm{status} + \mathrm{application}\ \mathrm{method} + \mathrm{equipment}\ \mathrm{repair}\ \mathrm{status}\right) \times \mathrm{P}\mathrm{P}\mathrm{E} $$

Mixing status was a two-level variable, based on never mixing and mixed (values of 0 and 9, respectively). Application method was a six-level variable, based on does not apply, use of aerial-aircraft, application in furrow, use of boom tractor, use of backpack, use of hand spray, and speed sprayer (values of 0, 1, 2, 3, 8, 9, and 9, respectively). Status of repairing equipment was two-level variable, based on not repairing and repairing equipment (0 and 2, respectively). PPE use was categorized as an eight-level variable based on the percentage of protection during pesticide spray. The pesticide exposure and cumulative exposure index were calculated as follows:

$$ *\ \mathrm{P}\mathrm{E}\mathrm{M} = \mathrm{pesticide}\ \mathrm{exposure}\ \mathrm{month} $$

$$ \mathrm{Spraying}\ \mathrm{year} \times \mathrm{spraying}\ \mathrm{day}\ \mathrm{per}\ \mathrm{year}\ /\ 30\ \mathrm{day} $$

$$ *\ \mathrm{C}\mathrm{E}\mathrm{I} = \mathrm{cumulative}\ \mathrm{exposure}\ \mathrm{index} $$

$$ \mathrm{Intensity}\ \mathrm{level} \times \mathrm{spraying}\ \mathrm{year} \times \mathrm{spraying}\ \mathrm{day}\ \mathrm{per}\ \mathrm{year} $$

### Pesticide metabolites

Organophosphorous pesticides were analyzed as metabolites of pesticides. When organophosphorous pesticide exposure occurs, it is metabolized via dealkylation, hydrolysis, and isomerization. Dialkyl-phosphate (DAP) in the urine can be used as a metabolite to indirectly measure organophosphorous pesticide exposure. The metabolites of DAP are measured as dimethylphosphate (DMP), diethylphosphate (DEP), dimethyl phosphorothioate (DMTP), diethyl phosphorothioate (DETP), dimethyl dithiophosphate (DMDTP), and diethyl dithiophosphate (DEDTP). In this study, 4 metabolites (DMP, DEP, DMTP, and DETP) among the 6 target metabolites were analyzed [16].

DMP (98%), DMTP (98%) and dibutylphosphate (DBP, 99%), used for an internal standard (I.S), was purchased from Acros Chimica. DEP (98%) was purchased from Supelco and DETP (98%), 2,3,4,5,6-pentafluorobenzyl bromide (derivatization reagent, 99.9%, PFBBr), toluene (99.5%) and n-hexane (99.9%) from Sigma Aldrich. Diethyl ether, acetonitrile, which are HPLC grade, hydrochloric acid (37%, HCl) and sodium sulfate anhydrous (99%, Na_2_SO_4_), sodium disulfite (Na_2_S_2_O_5_), potassium carbonate (K_2_CO_3_) were obtained from Burdick & Jackson. Gases used by the instrumentation had a minimum purity of 99.99%. Water used through out the experiments was distilled and deionized to 18MΩ with a Millipore Milli-Q system (Millipore Co., Bedford, MA, USA).

Electron ionisation mass spectrometric analysis was performed on a GC-MS Hewlett-Packard 6890 (gas chromatography) and HP 5973 mass spectrometer system equipped with a DB-5MS (30 m × 0.25 mm × 0.25um) capillary column. Gas (helium) with a column flow of 1 mL/min was used as a carrier gas. Injection volume was 2 μL of solution into the system in the splitless mode (split 20:1 at 1 min) and the column temperature was initially held at 80 °C for 1 min, raised to 250 °C at 20 °C/min, held for 10 min. The injector temperature was 250 °C. The ion source (detector) and interface temperatures were set at 280 °C and 300 °C, respectively. An auto-tune of the mass spectrometer using pentafluorotributylamin (PFTBA, tuning standard) was performed before the analysis.

DMP, DEP, DMTP and DETP were prepared at a concentration of 1000 mg/L in MeOH, and diluted with MeOH to each working standard solution at concentrations ranging from 3 to 100 mg/L. The standard solutions were stored in the dark at 4 °C. Five milliliters of urine was pipetted into a 15 mL screw top glass test tube, and 50 μL of internal standard solution (50 μg/L DBP), 5 g of NaCl, 1 mL of 6 M HCl, 50 mg of Na_2_S_2_O_5_, and 5 mL of diethylether- acetonitrile (1:1, v/v) were added.

After shaking for 5 min and vortex 1 min, the test tube was centrifuged (3000 rpm for 5 min). The organic phase (upper layer) containing DAP was transferred into a new screw-top glass test tube containing 15 mg K_2_CO_3_. The residuals were re-extracted with 5 mL of diethylether-acetonitrile (1:1, v/v) and then shaked for 5 min, vortexed, and centrifuged (3000 rpm for 5 min). The supernatant obtained from the second extraction was combined with the first extract. The resulting extract was evaporated at 45 °C to dryness with a gentle nitrogen stream. To the dried extracts, 15 mg K_2_CO_3_, 1 mL of ACN, and 50 μL of PFBBr were added and incubated in a water bath at 70 °C for 60 min with occasional swirling. Afterwards, 5 mL of water and 5 mL of n-hexane were added, and shaked the mixture for 5 min, vortexed, and centrifuged. The upper layer containing PFB-DAP was transferred to new test-glass tubes. The extraction was then repeated with 5 mL of n-hexane and the supernatant obtained from the second extraction was combined with the first extract. And extraction materials was evaporated at 45 °C to dryness with a gentle nitrogen stream. The residue was dissolved in 100 μL of toluene and injected into GC/MSD.

### Statistical analysis

SPSS 18.0 was used to evaluate the relationship between pesticide metabolite and oxidative stress. Demographic and lifestyle characteristics (i.e., age, smoking, drinking, and exercise), and pesticide characteristics (i.g., number of spraying years, number of spraying days per year, and spraying time), pesticide exposure index (PEM, CEI), and biomarker levels (i.e., 8-OHdG, MDA, isoprostane) were evaluated.

To better evaluate the relationship among 8-OHdG, MDA, isoprostane, and other variables, we used correlation coefficients. Linear regression analyses were used to assess the relationship between the levels of pesticide metabolites and oxidative stress biomarkers. Linear regression analyses adjusted for age, smoking, drinking, exercise. A *P* value < 0.05 was taken as being statistically significant.

Oxidative stress biomarkers and pesticide metabolites concentration were natural log-transformed to account for skewed distribution for linear regression models and logistic regression analysis.

## Results

### Quality control

The calibration curve of 8-OHdG, isoprostane, and MDA were produced at a range of 0.5 μg/L - 20 μg/L, 0.005 ng/mL - 1 ng/mL, 0.443 umol/L - 8.850 umol/L. The detection limit of 8-OHdG (0.053 μg/L), isoprostane (0.162 pg/mL) and MDA (0.0437 umol/L) was calculated by using the method proposed by the US EPA (Environmental Protection Agency). DMP, DEP, DMTP, and DETP were calculated as 0.84 μg/L, 1.35 μg/L, 0.59 μg/L, and 2.06 μg/L, respectively. The mean absolute recoveries ratio were 77.25–129.00% for DMP, 65.02–88.12% for DEP, 85.32–143.64% for DMTP, and 76.82–148.44% for DETP with pooled urine spiked with DAP.

### General characteristics and oxidative stress biomarker characteristics of the study subjects

Ages of subjects who are exposed to pesticides are 60-69, 44 persons out of the total subjects of 84 and that shows the highest level in the age groups. Regarding smoking, non-smoking and smoking subjects are investigated by 37 and 27 respectively. In the case of drinking, 55 subjects are addicted to drinking. Also, regarding exercises, 66 subjects have not been exercised (Table 1). The concentrations of 8-OHdG, Isoprostane, and MDA that represent oxidative stress biomarkers in subjects are investigated as 0.939 ± 0.61ug/g creatinine, 0.298 ± 0.18 ng/mg creatinine, and 0.133 ± 0.07umol/g creatinine, respectively (Table 2).

**Table 1:** Demographic characteristics of pesticide exposure farmer

	Pesticide exposed farmer
	*N* (%)
Demographic characteristics	
Age, n(%)	
~ 59	27 (32.2)
60 ~ 69	44 (52.3)
70~	13 (15.5)
Smoking, n(%)	
Non-smoking	37 (44.6)
Past-smoking	19 (22.9)
Smoking	27 (32.5)
Drinking, n(%)	
Non-drinking	22 (26.5)
Past-drinking	6 ( 7.2)
Drinking	55 (66.3)
Exercise, n(%)	
Non-exercise	66 (78.6)
Exercise	18 (21.4)

**Table 2:** Oxidative stress biomarker and pesticide metabolite characteristics of pesticide exposure farmer

	pesticide exposed farmer
	Mean ± SD
*Oxidative stress biomarker*	
8-OHdG, μg/g creatinine	0.939 ± 0.61
Isoprostane, ng/mg creatinine	0.298 ± 0.18
MDA, μmol/g creatinine	0.133 ± 0.07
*Pesticide characteristics*	
Farming duration	37.55 ± 14.08
Spraying year, year	24.17 ± 9.57
Spraying day per year, day	10.46 ± 6.68
PEM	6.02 ± 5.96
CEI	196.20 ± 241.26
*Pesticide metabolites (ug/g creatiine)*	
DMP	0.83 ± 0.89
DEP	1.48 ± 1.27
DMTP	3.24 ± 7.29
DETP	4.84 ± 8.31

*Abbreviations: PEM* pesticide exposure month (spraying year × spraying day per year /30 day)

*CEI* cumulative exposure index (intensity level × spraying year × spraying day per year)

*DMP* dimethylphosphate, *DEP* diethylphosphate

*DMTP* dimethylthiophosphate, *DETP* diethylthiophosphate

### Pesticide metabolite characteristics of pesticide exposed farmer

The engaged period of agriculture in subjects is presented as average 37 years. The period for spraying pesticides is average 24 years, and the average annual spraying period is about 10 days. In the results of the calculation of PEM based on using pesticide exposure days and months, it is 6.02, and the cumulative exposure index is 196.2. In the results of the measurement of DAP, which is known as an organophosphorous metabolic substance, the concentrations of DMP, DEP, DMTP, and DETP are 0.83ug/g creatinine, 1.48ug/g creatinine, 3.24ug/g creatinine, and 4.84ug/g creatinine, respectively (Table 2).

### Correlation analysis for oxidative stress biomarkers, pesticide exposure indices, and pesticide metabolites

In a simple correlation analysis, 8-OHdG showed a significant positive correlation with MDA (r = 0.471), CEI (r = 0.240), DMP (r = 0.285), DEP (r = 0.396), and DETP (r = 0.361) (p < 0.05). Isoprostane showed a significant positive correlation to MDA (r = 0.461), DMP (r = 0.484), DEP (r = 0.578), and DETP (r = 0.603) (p < 0.05). MDA showed a significant positive correlation to DMP (r = 0.532) and DEP (r = 0.506), and DETP (r =0.367) (p < 0.05). In addition, PEM, which is used as an index of pesticide exposure, showed a significant positive correlation to 8-OHdG (r = 0.326), isoprostane (r = 0.408), CEI (r = 0.771), and DMP (r = 0.280) (Table 3).

**Table 3:** Spearman’s Rank correlation coefficient between pesticide metabolite and biomarkers of oxidative stress

	8-OHdG	Isoprostane	MDA	PEM	CEI	DMP	DEP	DMTP	DETP
8-OHdG	1								
Isoprostane	0.187	1							
MDA	0.471**	0.461**	1						
PEM	0.326**	0.408**	0.254	1					
CEI	0.240*	0.226	0.133	0.771**	1				
DMP	0.285*	0.484**	0.532**	0.280*	0.096	1			
DEP	0.396**	0.578**	0.506**	0.186	0.102	0.723**	1		
DMTP	0.119	0.178	0.196	-0.045	-0.035	0.231	0.206	1	
DETP	0.361**	0.603**	0.367**	0.142	0.057	0.766**	0.895**	0.246*	1

*Abbreviations*: *CEI* cumulative exposure index (intensity level × spraying year × spraying day per year), *PEM* pesticide exposure month (spraying year × spraying day per year / 30 day)

*p < 0.05, **p < 0.01

*Abbreviation*: DMP (ug/g creatinine) ; dimethylphosphate, DEP (ug/g creatinine) ; diethylphosphate,

DMTP (ug/g creatinine) ; dimethylthiophosphate, DETP (ug/g creatinine) ; diethylthiophosphate

*PEM* Pesticide exposure month, *CEI* Cumulative exposure index

### Linear regression analysis of oxidative stress biomarkers and pesticide exposure

To investigate the influence of pesticide exposure on oxidative stress among farmers exposure to pesticide, a linear regression analysis was performed using 8-OHdG, isoprostane, and MDA as dependent variables with the application of a calibration for the demographic characteristics of age, smoking, drinking, and exercise.

The 8-OHdG level showed a significant relation to DMP (ß = 0.320, *p* = 0.020), DEP (ß = 0.390, *p* = 0.004), DETP (ß = 0.082, *p* = 0.015), and PEM (ß = 0.302, *p* = 0.020), and CEI (ß = 0.297, *p* = 0.020). In addition, isoprostane was related to DMP (ß = 0.396, *p* = 0.003), DEP (ß = 0.508, *p* = 0.000), DETP (ß = 0.504, *p* = 0.000), total DAP (ß = 0.302, *p* = 0.036), and PEM (ß = 0.434, *p* = 0.000).

MDA was related to DMP (ß = 0.432, *p* = 0.001), DEP (ß = 0.508, *p* = 0.000), and DETP (ß = 0.329, *p* = 0.014). Thus, the levels of the pesticide metabolites were significantly associated with the levels of oxidative stress biomarkers among farmers exposure to pesticide who were exposed to organophosphate pesticides (Table 4).

**Table 4:** Linear regression analysis of pesticide metabolites and oxidative stress biomarkers

Independent variables	8-OHdG (μg/g creatinine)*			Isoprostane (ng/mg creatinine)*			MDA (μmol/g creatinine)*		
	B	ß	*P*-value	B	ß	*P*-value	B	ß	*P*-value
DMP*	0.238	0.320	0.020	0.329	0.396	0.003	0.320	0.432	0.001
DEP*	0.306	0.390	0.004	0.471	0.508	0.000	0.426	0.508	0.000
DMTP*	0.009	0.019	0.897	-0.020	-0.051	0.732	-0.001	-0.002	0.987
DETP*	0.207	0.082	0.015	0.342	0.504	0.000	0.180	0.329	0.014
PEM	0.029	0.302	0.020	0.040	0.434	0.000	0.015	0.195	0.155
CEI	0.001	0.297	0.020	0.001	0.213	0.106	0.000	0.182	0.171

Adjusted : age, smoking history, alcohol drinking, exercise

*Oxidative stress biomarker and pesticide metabolites : log transformation

*Abbreviation*: DMP (ug/g creatinine) ; dimethylphosphate, DEP (ug/g creatinine) ; diethylphosphate,

DMTP (ug/g creatinine) ; dimethylthiophosphate, DETP (ug/g creatinine) ; diethylthiophosphate

*PEM* Pesticide exposure month, *CEI* Cumulative exposure index

## Discussion

Pesticides increase oxidative stress, leading to an imbalance between oxidants and antioxidants [17]. It has been reported that organophosphorus pesticides disturb the cytochrome p 450 system in the liver and affect the transfer system related to mitochondrial membranes [18]. In addition, it has been known that organophosphorus pesticides cause oxidative stress by inhibiting enzymatic and nonenzymatic antioxidant defenses [19, 20]. Other studies have shown that acute and chronic pesticide exposure affect internal secretions and the pancreas [21, 22].

And organophosphorous pesticide are neurotoxic in nature by acting as inhibitor of neuronal cholinesterase activity. There are numerous reported studies suggesting that organophosphorous pesticide caused lipid peroxidation [23]. Organophosphorous pesticide may induce oxidative stress leading to generation of free radicals and alterations in antioxidant or reactive oxygen species scavenging enzymes [24, 25]. We determined dialkylphosphate, which is considered to be a marker for pesticide metabolite and pesticide exposure index.

In this study, oxidative stress biomarkers, such as 8-OHdG, isoprostane, and MDA, were measured in subjects with and pesticide metabolite. The PEM (spraying years × spraying days per year/30 days), CEI (intensity × spraying years × spraying days per year), and DAP levels (DMP, DEP, DMTP, and DETP) as well as PEM were positively correlated to the levels of 8-OHdG, isoprostane, CEI, and DMP. CEI, DMP, DEP, and DETP also represented a positive correlation to 8-OHdG (Table 3).

The MDA and isoprostane levels directly represent lipid peroxidation resulting from exposure to organophosphorus pesticides. Thus, our findings are consistent with those of other studies in which the oxidative stress was increased by the toxicity of synthetic. pyrethroid, organochlorine, and carbamate pesticides, including xenobiotics [26, 27].

In the linear regression analysis (Table 4), a correlation analysis was performed after demographic characteristics, such as age, smoking, drinking, and exercise, were adjusted to determine the relationship between pesticide exposure and oxidative stress. As a result, 8-OHdG, isoprostane, and MDA were significantly related to DMP (ß = 0.320, *p*= 0.020), DEP (ß = 0.390, *p* = 0.004), and DETP (ß = 0.082, *p* = 0.015); DMP (ß = 0.396, *p* = 0.003), DEP (ß = 0.508, *p* = 0.000), and DETP (ß = 0.504, 0.000); and DMP (ß = 0.432, *p* = 0.001), DEP (ß = 0.508, *p* = 0.000), and DETP (ß = 0.329, *p* = 0.014), respectively. Organophosphorus pesticide mediated formation of ROS that activate protein kinase. Organophosphorus pesticide generated ROS formation and oxidative stress have been shown to be associated with apoptosis in different tissues [23, 28].

Thus, pesticide exposure should be controlled for farmers and the population at large. It is necessary to strictly control the access to pesticides, to minimize their use, and to replace highly toxic pesticides with those of low toxicity.

Prospective studies are required to verify the causal relationships between pesticide exposure and chronic diseases. In addition, it is necessary to consider the application and exposure characteristics of the pesticides and to exactly reproduce previous pesticide exposures [27]. Moreover, further studies are required in a larger farmer population to ensure statistical power, and a proper measurement of the biomarkers is required to reduce misclassification biases in the measurements of pesticide exposure and its influences on health.

The major limitation of this study is the cross-sectional design. Some of the results were not statistically significant because of the small size.

## Conclusions

The concentration between oxidative stress biomarkers and the pesticide metabolite were a positive correlation. Indicators of oxidative stress was associated with a pesticide metabolite DMP, DEP, and DETP. Therefore, Pesticide exposure and oxidative stress were relevant.
